# A novel hybrid face mask detection approach using Transformer and convolutional neural network models

**DOI:** 10.7717/peerj-cs.1265

**Published:** 2023-03-27

**Authors:** Haifa M. Al-Sarrar, Heyam H. Al-Baity

**Affiliations:** Information Technology Department, Collage of Computer and Information Sciences, King Saud University, Riyadh, Saudi Arabia

**Keywords:** Artificial Intelligence, Computer vision, Deep learning, Transformers, CNN, Face mask detection

## Abstract

Face and face mask detection are one of the most popular topics in computer vision literature. Face mask detection refers to the detection of people’s faces in digital images and determining whether they are wearing a face mask. It can be of great benefit in different domains by ensuring public safety through the monitoring of face masks. Current research details a range of proposed face mask detection models, but most of them are mainly based on convolutional neural network models. These models have some drawbacks, such as their not being robust enough for low quality images and their being unable to capture long-range dependencies. These shortcomings can be overcome using transformer neural networks. Transformer is a type of deep learning that is based on the self-attention mechanism, and its strong capabilities have attracted the attention of computer vision researchers who apply this advanced neural network architecture to visual data as it can handle long-range dependencies between input sequence elements. In this study, we developed an automatic hybrid face mask detection model that is a combination of a transformer neural network and a convolutional neural network models which can be used to detect and determine whether people are wearing face masks. The proposed hybrid model’s performance was evaluated and compared to other state-of-the-art face mask detection models, and the experimental results proved the proposed model’s ability to achieve a highest average precision of 89.4% with an execution time of 2.8 s. Thus, the proposed hybrid model is fit for a practical, real-time trial and can contribute towards public healthcare in terms of infectious disease control.

## Introduction

In the last decade, there has been a large increase in the number of digital image and video databases due to the rapid evolution of technology. To make sense of this huge number, the automatic understanding of information by intelligent computer vision technologies is needed as manual methods have become difficult to process. Computer vision enables computers to process digital images and videos automatically, thereby allowing them to perform specific tasks and derive meaningful information (Dickson, 2019).

One of the most challenging tasks in the field of computer vision is object detection, which entails identifying the class of objects in digital images by predicting each object’s bounding boxes and category labels (Liu et al., 2020). Face detection is part of the object detection field, and it is popular subject in computer vision literature. It is attracting widespread interest due to the immense number of applications that require automatic facial image analysis as a first step (Rath & Rautaray, 2014). With recent developments in deep learning (DL) models, face detection processes have been enhanced to detect several face variations such as occluded faces, which has also been called face mask detection. The process of detecting face masks is more difficult than detecting normal faces due to the lack of some facial features, such as the nose, mouth, and chin, which are covered by face masks.

Face mask detection can be very beneficial in different domains as it can be used to ensure public safety. For example, face mask detection technology can be employed in operating rooms to ensure that healthcare personnel are wearing their mandatory face masks, and it can be used in locations where chemical emissions are expected, such as factories, or in situations of hazy or dusty weather, which is a common occurrence in Saudi Arabia. Moreover, COVID-19 pandemic recently made face mask detection a critical issue.

At the beginning of 2020, the World Health Organization (WHO) declared the COVID-19 infection a global pandemic. A study conducted by Kwekha-Rashid, Abduljabbar & Alhayani (2021) stated that COVID-19 was rapidly being transmitted between people, causing 73,806,583 infections with more than 1,641,635 reported deaths by the end of 2020. In response to these developments, governments mandate the wearing of face masks recommendations by medical experts in order to limit the spread of COVID-19 and mitigate its risks. Therefore, to avoid the tedious and difficult task of manually monitoring people’s behavior in public places, accurate face mask detection technology needs to be adopted to automatically detect whether people are wearing the required face masks.

The research on face mask detection has recently gained much attention, especially after the COVID-19 pandemic. Current detection solutions mainly focus on building DL-based models using convolutional neural network (CNN) algorithms, such as you only look once (YOLO) and single-shot detector (SSD), which performed better than machine learning (ML) models (Nowrin et al., 2021). However, it is still a problem, and further improvements can be made to enhance the speed and accuracy of classification.

Transformer is a new type of deep neural network proposed by Vaswani et al. (2017) that was developed primarily on the basis of the self-attention mechanism. It was first used in the natural language processing domain but in recent years, transformer’s strong capabilities in a wide range of natural language processing tasks have attracted the attention of computer vision researchers who have applied this advanced neural network architecture to visual data. Transformer has shown competitive or improved performance compared to other networks, such as the CNN, in the computer vision field, and it has gained much interest due to its tremendous capabilities. Transformer is able to capture long-range dependencies within visual inputs, which improves the utilization of the graphical processing unit, and increases its training efficiency, and improves its scalability to complex models and very large datasets (Khan et al., 2021).

Inspired by transformer’s powerful representation capabilities and its very promising results in the computer vision domain, we propose extending transformer by including face mask detection tasks. To the best of our knowledge, no previous studies have tackled the problem of face mask detection using the current advanced transformer models. In other words, the classification accuracy can still be improved. In this study, we bridge this gap by developing a novel hybrid automatic model for face mask detection and classification problems using a transformer-CNN-based approach for the purpose of enhancing the speed and accuracy of classifications. Consequently, this study contributes to public healthcare by avoiding the traditional and tedious process of manually monitoring people’s behavior for infection control. Moreover, this study will contribute to the exploration to this proposed model’s efficiency by answering the following research question: To what extent can the transformer-CNN hybrid model provide satisfactory performance in terms of face mask detection accuracy as compared to other state-of-the-art network models?

Face mask detection comprises two main stages: face detection and face mask classification. The first stage consists of detecting faces in the input image, and the second stage entails classifying whether the detected faces are wearing face masks (Nowrin et al., 2021). As detection and classification are considered two different tasks, two different models were constructed for each stage herein. After this, these two models were combined in the final hybrid model, which performed face mask detection. The detection and the classification models’ performances were evaluated separately through different experiments to ensure the final hybrid model’s robust performance. The detection transformer (DETR) algorithm was used as the theoretical framework for the detection stage, and the AlexNet CNN algorithm was the theoretical framework for the classification model.

The rest of this paper is organized as follows: “Related Work” presents an overview of the existing research on face mask detection; “Materials and Methods” describes the study’s methodology; “Experimental Evaluation” details the experiments and discusses the evaluation results; and “Conclusion and Future Work” concludes the article and presents potential future avenues of research.

## Related work

Many studies have been conducted on face detection in the last few years, and they have been discussed intensively due to their wide range of applications, with many face mask detection studies being conducted after 2020 due to the outbreak of COVID-19.

Face mask detection algorithms have been developed using object and face detection techniques. As detecting face masks is the detection of a face mask on person, it is closely related to detecting a specific class of objects in a given image, which are faces in this study (Kumar, Kaur & Kumar, 2019; Wang et al., 2020). There are few studies on face mask detection compared to other computer vision tasks. The following sections will discuss the existing research on face mask detection in detail, which are divided into three categories according to the approaches used: ML-based approach, DL-based approach, and hybrid-based approach.

### ML-based approach

Traditional ML techniques are usually based on handcrafted feature extraction, but only two studies have used ML algorithms to develop detection mechanisms: the first is by Nieto-Rodríguez, Mucientes & Brea (2015); and the second is Ejaz et al.’s (2019) study.

In the first one, Nieto-Rodríguez, Mucientes & Brea (2015) propose a real-time face mask detection system for detecting if healthcare personnel are wearing face masks in an operating room for which a face detector and mask detector were combined. The approach was mainly based on the Viola-Jones algorithm, which was used to detect the presence of faces in any given image, and LogitBoost, which is a variant of the AdaBoost algorithm, to detect the presence of surgical masks on healthcare professionals’ faces, with each of the detectors having a color filter. However, these detectors would give false detection results if clothing was present next to the masked area due to their dependency on color filtering. This problem was overcome using synthetic rotation. The Labeled Faces in the Wild dataset (Huang et al., 2008) was used in the training stage, and Bao dataset was used for the testing stage. The proposed system triggered an alarm when the healthcare personnel did not wear the required face mask, and it had a recall rate above 95% and a false positive rate under 5%.

Ejaz et al.’s (2019) study proposed a face mask detector that was based on a traditional ML approach: After obtaining the image, the Viola-Jones algorithm was applied to detect the face region. To achieve the best results, some pre-processing methods were implemented to normalize and enhance the images of facial regions using matrix laboratory (MATLAB) toolboxes. After that, principal component analysis algorithm was applied to extract features from these pre-processed input images to identify if the face in the image was masked or non-masked. This system used the ORL Database of Faces (Our Database of Faces) (AT&T Laboratories, 1994). The experimental results showed that the proposed methodology was more effect for normal face detection but not for masked face detection since it had an average accuracy of 72% for masked face image detection but a 95% accuracy rate for normal face or non-masked face image detection.

The application of traditional ML techniques to face mask detection is rare, and the results are not promising. Therefore, most of the studies on face mask detection have employed DL-based approaches.

### DL-based approach

Most of the current algorithms used for face mask detection are DL-based models as they are more effective than traditional ML-based approaches. DL-based approaches have advanced more than any other approach in the field of computer vision since DL-based algorithms can be used to determine more about image data and because they can extract features automatically due to their incorporating many neural network layers in the processing stage. Therefore, we focused on a DL-based approach, which has been largely used in the literature on face mask detection.

The CNN family is one of the most widely used DL-based algorithms in computer vision studies, even though these CNN-based algorithms have some drawbacks, such as their not being robust enough for low quality images and their being unable to capture long-range dependencies.

The Masked Faces (MAFA) dataset is one in which any type of occlusion of the face, not only masks, is determined to be a mask. This dataset is one of the most popular datasets for training face mask detectors, and it is publicly available online. Using this MAFA dataset, Ge et al. (2017) designed a locally linear embedding (LLE)-CNN algorithm for face mask detection, which was divided into three major modules: a proposal module; an embedded module; and a verification module. The proposed module used two pre-trained CNN models for facial proposal generation and feature extraction, respectively: P-Net (Zhang et al., 2016) and Visual Geometry Group–Face (Parkhi, Vedaldi & Zisserman, 2015). The LLE algorithm was integrated into the embedded module to recover the facial cues that were missed due to different types of occlusions, including face masks. The last module performed the aggregation and classification tasks using unified CNN to identify whether it was a real face or not. This framework was compared to six state-of-the-art face detection models, and it outperformed them with an average precision (AP) of 76.4%.

An automatic facemask-wearing condition identification algorithm was designed by (Qin & Li, 2020), who combined the image super resolution (SR) network and the classification network to create the SRCNet. The former was used to process faces with low-resolution, and the later was used to separate the input face images into three categories: “no facemask-wearing;” “incorrect facemask-wearing;” and “correct facemask-wearing.” This method was used to identify face mask wearing conditions in order to distinguish between the faces that were wearing the face mask correctly and those that were not. The first step in the proposed algorithm entailed pre-processing the input raw images using MATLAB toolboxes to improve the accuracy for the subsequent detection and classification steps. After this, the multitask cascaded CNN algorithm was applied as the face detector to obtain and crop the facial areas. If the cropped images were less than 150 p in size, they served as inputs in the SR network to ensure higher quality images, after which they were analyzed in the facemask-wearing condition identification step. If the images were higher than 150 p, the cropped images were sent directly to the facemask-wearing condition identification. The last step of this algorithm was image classification for which MobileNet-V2 was adopted to classify the input images as either “no face mask wearing,” “incorrect face mask wearing,” or “correct face mask wearing.” The proposed model was trained and tested using the Medical Mask Dataset (MMD) that is publicly available on (Waghe, 2020) and it reported a 98.70% accuracy rate.

Jiang, Fan & Yan (2020) proposed a novel DL-based face mask detector called RetinaFaceMask that is capable of detecting if a person is wearing a face mask or not. Inspired by RetinaNet (Lin et al. 2018), this model used a detection network that consisted of a backbone, neck, and head. The ResNet (He et al., 2016) and MobileNet (Howard et al., 2017) models were adopted as the backbone and were used to extract information from images in the form of feature maps. To connect the backbone and the head, feature pyramid network was adopted as a neck to enhance the original feature map in order to ensure advanced precision. Finally, the head of the model refers to a classifier or detector similar to that used for SSD in which a novel context attention module was added to improve the detection of faces with and without masks. The model was trained and tested using Face Mask Dataset (FMD) compromising the WIDER Face dataset (Yang et al., 2016) and the MAFA dataset (Ge et al., 2017), and it achieved promising results in terms of precision and recall metrics. However, it is important to pinpoint that face detection was measured separately from mask detection, resulting in these impressive results.

Similar to Qin & Li’s (2020) study, Inamdar and Mehendale (Inamdar & Mehendale, 2020) developed a DL-based model called Facemasknet for the purpose of identifying if people were wearing face masks properly and classifying them into categories: “with a mask,” “improperly worn mask,” and “without a mask.” This model was trained and tested using a customized dataset that contained 35 images and video streams of individuals wearing a face mask properly or improperly and individuals not wearing masks. This dataset is not publicly available. The inputs were loaded into the model, after which the two detectors were used: The first detected the faces and extracted regions of interest, after which the detector was applied to categorize the input data into the three groups. Green and yellow bounding boxes were used to localize the detected faces and masks respectively. This proposed model was built using MATLAB and had an accuracy rate of 98.6% compared to similar models.

Batagelj et al. (2021) introduced a DL-based model similar to that used by Qin & Li (2020) as both were aimed towards detecting not only the presence of masks but also whether face masks are worn correctly. This study introduced a novel annotation dataset of facial images called the Face-Mask Label Dataset, which is available for public use. This dataset was compiled from the publicly available WIDER Face and MAFA datasets. A complete system comprising two stages was designed. The first stage entailed detecting faces in the input images using the CNN-based RetinaFace model (Deng et al., 2020) and the second stage consisted of face classification to determine if people were wearing the face masks correctly or not. All of the detected faces were classified as “compliant” or “non-compliant” based on the mask placement using the ResNet-152 model (Simonyan & Zisserman, 2015). The proposed approach performed better than all of the existing baseline models, with AP scores of 81.3% and 87.1% at Intersection over Union thresholds of 50% and 40%, respectively.

Loey et al. (2021b) proposed a novel deep learning-based DL-based model for face mask detection that used an updated version of an algorithm from the YOLO family, YOLOv2, and the ResNet-50 model. This model consisted of two components: The first was designed to extract features from input images using the ResNet-50 model; and the second was designed to detect the face masks using YOLOv2. The proposed model was trained and tested using a combination of the MMD and the FMD. A comparative study was also presented in the paper. The proposed model had better performance than the previous methods as its AP reached 81%.

Nagrath et al. (2021) proposed SSDMNV2 for face mask detection which is capable of real-time mask detection since it can be embedded in devices, such as Raspberry Pi or surveillance cameras. The proposed algorithm used the Single Shot Multibox detector’s object detection model (Liu et al., 2016) in combination with ResNet-10 (Anisimov & Khanova, 2017) as a backbone to detect faces. MobileNetV2 was applied as a classifier in the SSDMNV2 to determine whether a person was wearing a face mask by predicting the presence of the mask. This model was trained and tested using a dataset that was a combination of publicly available datasets, including the MMD (Waghe, 2020) and the dataset provided by Wang et al. (2020). This dataset is available on GitHub for public use. This proposed SSDMNV2 model was compared to various existing models trained on the same dataset, and the comparison showed that this model outperformed all other models, with an accuracy rate of 92.64% and an F1 score of 0.93.

### Hybrid-based approach

As ML-based approaches are not effective for face mask detection, most researchers use DL-based or hybrid-based approaches, which combine both DL-based and ML-based approaches.

A few studies have adopted the hybrid-based method. For instance, Ristea & Ionescu (2020) improved upon an existed hybrid-based face mask detection approach in order to detect whether a person is wearing a face mask according to their speech. This approach comprised two steps: (1) training generative adversarial network on cycle-consistency loss, which transformed the unpaired utterance between the “with-mask” and “without-mask” classes; and (2) assigning the opposite labels to each transformed pronunciation and creating new training accents by using a cycle-consistent generative adversarial network. The initial and transformed utterances were converted into spectra and then used as inputs in multiple ResNet neural networks (He et al., 2016) that had different depths. The networks were then combined using classifier support vector machines (SVMs). ResNets were used to produce feature vectors and were then concatenated and used as inputs for the SVMs, which predicted the final results of “mask” or “no mask”. The dataset used in this study was provided by the ComParE organizers, and the model had an accuracy of 76.4%.

Loey et al. (2021a) proposed a face mask detection model that consisted of two main components: The first was designed for feature extraction; and the second for classification. As a feature extractor, the ResNet-50 model was applied and classical ML algorithms like SVM, decision tree, and ensemble were used as the proposed model’s classifiers. The model was trained, validated, and tested using the Real-World Masked Face Dataset, the Simulated Masked Face Dataset, and Labeled Faces in the Wild dataset (Learned-Miller et al., 2016). The SVM classifier was used to ensure the highest performance and shorter time periods than other classifiers in the training process, and it achieved an accuracy rate of 99.64%.

Table 1 summarizes these previous studies on face mask detection in the last two years.

**Table 1:** Summary of the previous works.

Article	Approach	Dataset	Results	Shortcomings
Nieto-Rodríguez, Mucientes & Brea (2015)	Machine learning	1. LFW 2. BOA	Recall = above 95% False positive rate = under 5%	1) Limited to detect surgical type of mask face in the operation room. 2) Low performance because of using the color filters. 3) Using shallow ML approach.
Ejaz et al. (2019)		ORL face	Accuracy = Non-masked faces: 95% Masked faces: 72%	1) Low performance of face mask detection. 2) Using shallow ML approach.
Ge et al. (2017)	Deep learning	1. AFLW 2. MAFA	Average precision = 76.4%	1) The used dataset includes any type of face occlusion such as human body or other objects which is not the focus of this study.
Qin & Li (2020)		MMD	Accuracy = 98.70%	1) Slow detection speed. 2) Small dataset.
Jiang, Fan & Yan (2020)		Face mask dataset	Precision = With mask: 93.4% Without mask: 91.9% Recall = With mask: 94.5% Without mask: 96.3%	1) Small dataset. 2) High computation overhead because of the use of ResNet. 3) The detection results of masked faces calculated separately of the normal faces, which may lead to higher performance than if mixed.
Inamdar & Mehendale (2020)		Not available	Accuracy = 98.6%	1) Very small and biased dataset. 2) The used dataset is not publicly available.
Batagelj et al. (2021)		FMLD	Average precision = IoU thresholds of 50%: 81.3% IoU thresholds of 40%: 87.1%	1) The purpose is to detect if the mask is worn correctly or not which is not the focus of this study.
Loey et al. (2021b)		1. MMD 2. FMD	Average precision = 81%	1) Relatively small dataset.
Nagrath et al. (2021)		1. MMD 2. RMFD	Accuracy = 92.64% F1-score = 0.93	1) The measured performance is for the social distancing detection which is not the focus of this study.
Ristea & Ionescu (2020)	Hybrid	Mask Augsburg Speech *Corpus*	Accuracy = 76.4%	1) The used dataset is audio samples for audio detection, which is not the focus of our study.
Loey et al. (2021a)		1. RMFD 2. SMFD 3. LFW	Accuracy = 99.64%	1) Using shallow ML approach in the classification stage. 2) The dataset that achieved high results is composed of only normal face objects, *i.e*. without any face mask object.

These studies include models that perform well, especially those that adopted the DL CNN-based algorithm. However, these studies have some limitations as they used relatively small datasets due to the lack of rich face mask benchmark datasets. This resulted in biased decisions being made in addition to poor generalizability about the models. However, some of the CNN algorithms used in these studies require advanced computation, which could lead to longer processing times slower detection speeds or worse performance. Therefore, there is still a need for high detection speeds of face masks as well as for these models to perform powerfully and accurately.

Since transformer-based models have not yet been applied to face mask detection and have shown successful results when used in the object detection field, this study explores the transformer neural network’s capabilities in ensuring competitive or even better performance than the existing methods in terms of face mask detection. Therefore, in this study, we developed an automatic hybrid face mask detection model that combined transformer and CNN algorithms to overcome the shortcomings of the algorithms used in the previous studies. An AlexNet CNN-based model (Krizhevsky, Sutskever & Hinton, 2012) and a DETR transformer-based model (Carion et al., 2020) were used to develop the proposed hybrid model.

## Materials and Methods

**Figure 1:**
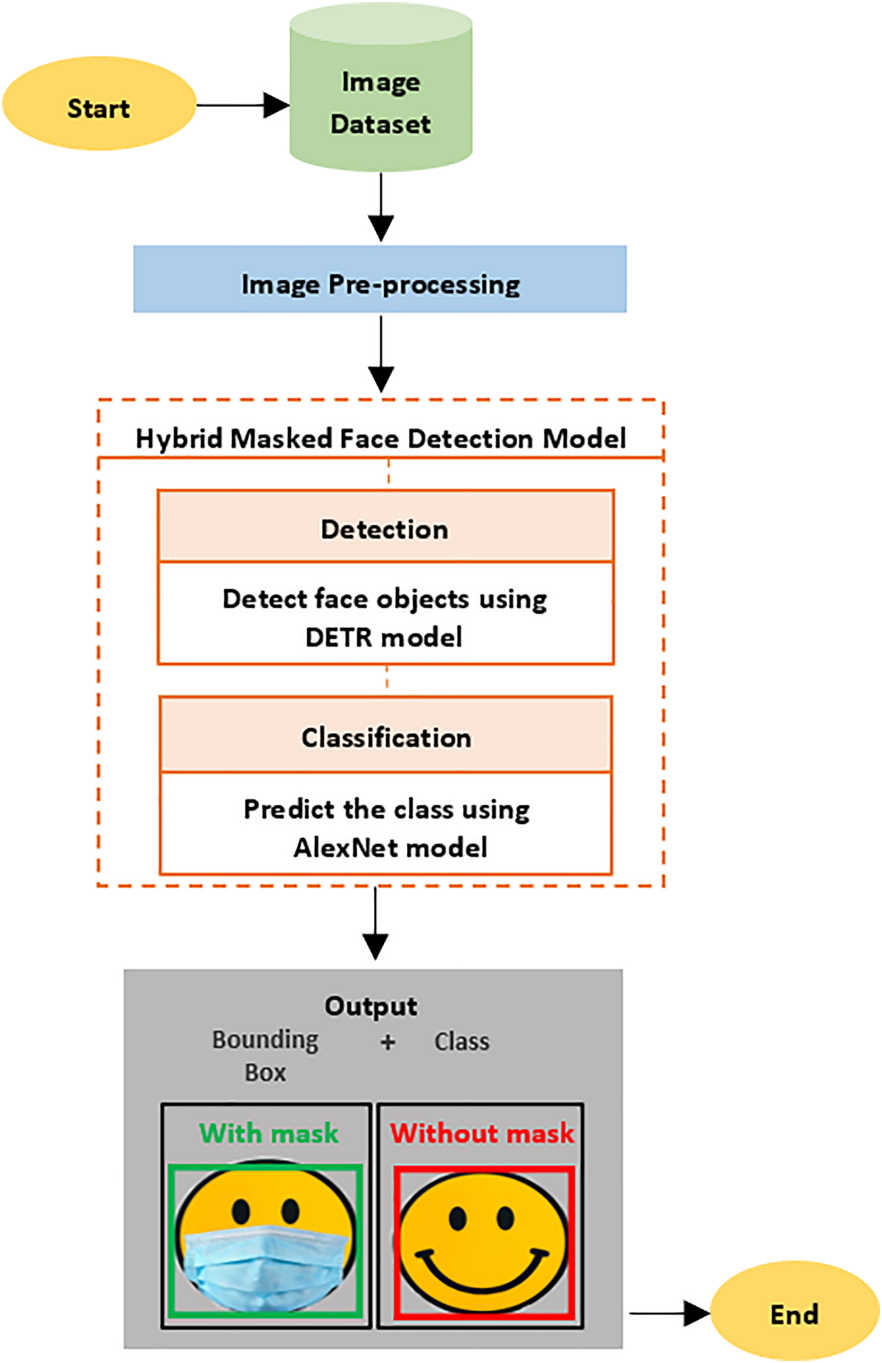
The proposed model framework.

The model used in this study is a combination of transformer and CNN approaches, which were merged to increase detection efficiency. The proposed model detects faces in digital images, which are then classified according to whether or not face masks are being worn. There are several steps that are followed in the proposed model’s framework to achieve the goal of this research. These steps are: 1) obtaining the image datasets; 2) pre-processing the data; 3) building the proposed model; and 4) evaluating the model (see Fig. 1).

### Dataset

Generally, the implementation of DL models requires a massive dataset. Many datasets have been constructed and made available for face detection and recognition, but there are only a few face mask detection datasets that are publicly available, which is one of the major challenges for detecting face masks. Due to this scarcity of rich datasets and to avoid issues with generalizability, two datasets were combined to train and test the proposed model: The FMD (AIZOO) (Chiang & Hongliang, 2020; https://github.com/AIZOOTech/FaceMaskDetection) and the MMD (Waghe, 2020).

The FMD or AIZOO is a public open-source dataset that contains 7,959 images and more than 15,000 annotated face objects. Each image’s annotation file is in XML format that illustrates the class label, size, and the bounding box coordinates.

The MMD is one of the most effective datasets for face mask detection. It consists of 682 images that each have associated XML annotation files that specify whether the faces in the images are wearing masks, in addition to the image size and the bounding box coordinates. In total, the MMD includes more than 3,000 objects. It is published by Waghe (2020).

**Figure 2:**
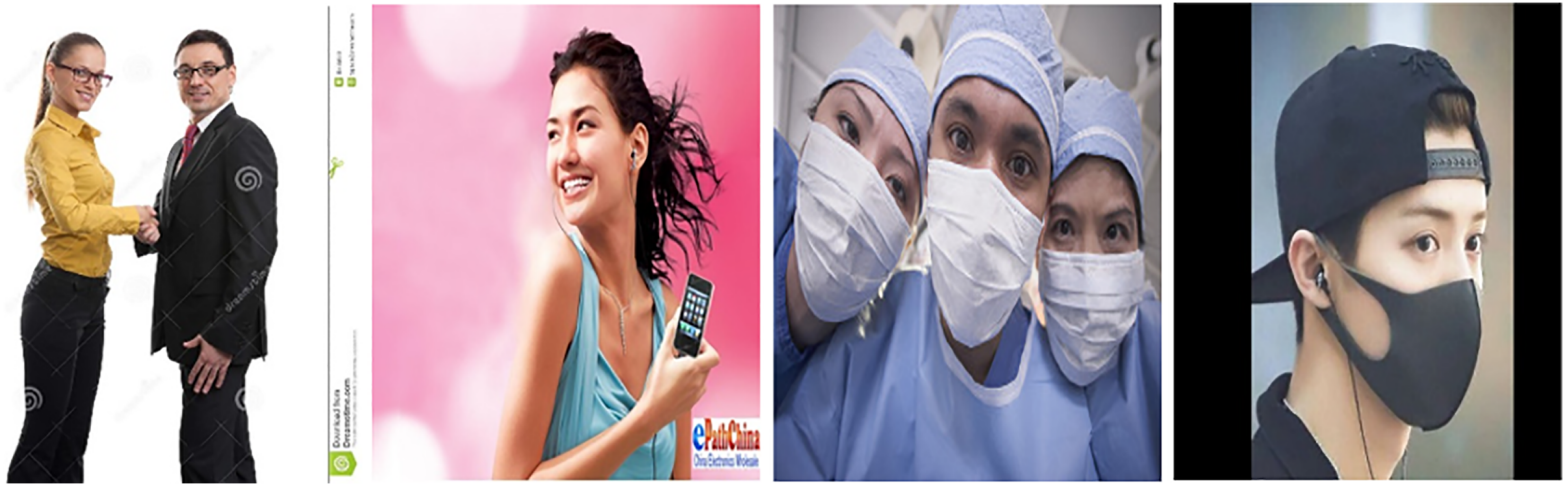
Sample images from the dataset. Image source credits: WIDER FACE database (http://shuoyang1213.me/WIDERFACE/index.html, MIT license) and MAFA database (https://www.kaggle.com/datasets/revanthrex/mafadataset, Apache-2.0).

These datasets were combined to obtain a final dataset comprising approximately 9,000 images with more than 18,000 annotated face objects (see Fig. 2 for sample images from this final dataset).

### Data prepocessing

Since the datasets have different annotation file formats with different class labels, it was important to align the annotation files to our pre-trained models and to make all of the datasets compatible by ensuring the same annotation format. Therefore, we created a comma-separated values annotation file for each dataset, which contained the same input elements.

Additionally, the input images needed to be made suitable for the models to improve the accuracy of the subsequent detection and classification steps. Hence, a resizing technique was applied to the data. Image resizing is one of the most critical pre-processing techniques in computer vision, and it was applied to increase the proposed model’s training speed. Thus, the images were resized to 215 × 215 p to make them suitable as inputs for the pre-trained DETR model. The DETR model was trained using the COCO dataset, and our dataset was pre-processed to match this format. Following that, each image was resized to 224 × 224 p to make it suitable as an input for the pre-trained AlexNet model, which was trained using the ImageNet dataset, and our dataset was pre-processed to match this dataset’s format.

Another important technique was employed: image augmentation. This is a common technique for expanding the dataset by applying multiple image transformations to the data, such as zooming, flipping, and rotation, to make this data appear different to a machine. This technique was used to improve the model’s robustness and our ability to generalize the results as well as to avoid overfitting.

### The algorithms used

DETR is a transformer-based model that is used for object detection (Carion et al., 2020) as it handles object detection as a direct set prediction problem and proposes a set of loss function. This means that when a set of image features enters the transformer’s encoder-decoder, it will predict a set of object classes and bounding boxes for up to 50 objects at once. In our proposed model, DETR was employed as the backbone to detect all of the faces in any given digital image, with a bounding box surrounding these faces.

Additionally, the pretrained CNN AlexNet model (Krizhevsky, Sutskever & Hinton, 2012) was used in the classification stage of our proposed model due to CNN’s good performance in image classification tasks. AlexNet was the first CNN-based model that utilized graphics processing unit to boosts its performance. Moreover, AlexNet has a very simple architecture, which was of great benefit in this study because it led to faster classification speeds, which is the aim of this research.

AlexNet was employed as the head in our proposed model to classify the faces that were detected in the prior detection stage. To achieve this study’s aims, the detected faces were then divided into one of two classes: “with mask;” and “without mask.”

### Building the proposed model

The proposed hybrid DL model was developed using the DETR model for the detection stage and the AlexNet model for the classification stage. Firstly, the DETR detection model was trained to produce bounding boxes around the detected faces. Secondly, the AlexNet classification model was trained to produce a label of either “with mask” or “without mask” for the detected faces. Finally, these two models were combined to perform the whole task by feeding the results of DETR model into the AlexNet model. The detection and the classification models were built and evaluated separately by applying different experiments to ensure the final hybrid model’s robust performance. These models were trained and tested using the combination of AIZOO and the MMD.

To decide on the best split ratio for the dataset, three split ratios of 80/20, 70/30, and 60/40 were considered. A separate experiment was conducted on each split ratio for each model in order to determine the optimum split ratio.

## Experimental evaluation

To evaluate the performance of the proposed hybrid model, two experiments were conducted: (1) the initial experiment for creating the detection model based on DETR and for creating the classification model based on AlexNet using the training set, after which their performance was evaluated their performance using the testing set; and (2) The proposed hybrid face mask detection model experiment, which combined the detection and classification models.

### The initial experiment

Different experiments were performed to evaluate the proposed detection and classification models’ performances in order to ensure the final model’s robust performance. This experiment a preliminary step for the hybridization of the models.

#### The detection model (DETR)

Initially, the dataset was tested using three split ratios: 80/20, 70/30, and 60/40. Separate experiments were conducted for each split ratio to determine the optimal split. The results for each split ratio in terms of loss value demonstrated that the 60/40 split ratio had the best performance (see Table 2).

**Table 2:** Results of dataset splits for DETR detection model. Bold indicates the model best result.

Split ratio	80/20	70/30	**60/40**
Loss	0.741	0.519	**0.491**

Additionally, the tuning process for the hyperparameters, such as batch size, number of epochs, and learning rate, was essential in building the proposed model as it played a crucial role in the proposed model’s prediction accuracy due to it having a direct impact on how well the model was trained. In order to ensure the best results for the model, the tuning process was used to identify the optimal values for the hyperparameters. The learning rate was set to a value of 0.00002, and if a larger learning rate value was used, the loss rate would have stopped decreasing after a number of epochs, thereby disrupting the training process (Smith, 2018). As for the number of epochs, 20 was selected when the loss value stopped decreasing. In each epoch, a batch size of 30 was used since the optimal batch size in ML model’s learning processes are typically approximately 30 to 64 (Kandel & Castelli, 2020).

For the optimization method, the optimizer considered an algorithm that can be used to enhance the neural network’s attributes, such as learning rate and weights, in order to decrease the losses (Nowrin et al., 2021). Adam, AdamW, and SGD optimizers were tested, with Adam providing the best results (see Table 3).

**Table 3:** Results of the used optimizers in DETR detection model. Bold indicates the model best result.

Optimizers	Loss
Adam	**0.379**
AdamW	0.462
SGD	0.491

As the DETR model adopted end-to-end set predictions for object detection, cross entropy loss function was used to evaluate the predicted and truth value of the face objects. Equation (1) expresses the DETR loss function that was used in the DETR in order to compute the loss for the matched objects’ pairs.

Equation (1) DETR loss equation (Han et al., 2022).

(1) $${{\rm {\cal L}}_{Hungarian}}\; \; \left( {{\rm y,}\hat y} \right) = \; \mathop \sum \limits_{i = 1}^N \left[\; - \; \log {\hat p_{\; \hat \sigma }}_{\left( i \right)}\left( {{c_i}} \right) + \; {1_{\left\{ {{c_i} \ne \emptyset } \right\}\; }}{{\rm {\cal L}}_{box}}\left( {{b_i},{{\hat b}_{\hat \sigma }}\left( i \right)} \right)\right]$$

where:

-
$\hat \sigma$ = the optimal assignment.
-
${c_i}$ and ${\widehat {p{\rm \; }}_{\hat \sigma \left( i \right)}}\left( {{c_i}} \right)$ = the target class and predicted class labels, respectively.
-
${b_i}$ and ${\hat b_{\hat \sigma }}\left( i \right)$ = the truth value and the predicted bounding box.
-
$y = \left\{ {\left( {{c_i},{b_i}} \right)} \right\}$ and $\hat y$ = the truth value and the prediction of objects.

#### The classification model (ALEXNET)

Similar to the DETR model, the dataset was examined to determine the best split ratio for the AlexNet model, with an 80/20 split ratio being selected in this model since it achieved the minimum loss value (see Table 4). The tuning process for the hyperparameters was also performed for this model. The employed learning rate was set to a value of 0.001, which gave the best performance as a lesser learning rate might have increased the overfitting risk. Adam, AdamW, and SGD were the optimizers that were tested, and the latter provided the best results (see Table 5). Cross entropy loss function was also used in the AlexNet model. Regarding the number of epochs and batch size, 10 epochs were selected because the loss value started to increase after an epoch value of 10, and a batch size of 50 was used.

**Table 4:** Results of dataset splits for AlexNet classification model. Bold indicates the model best result.

Split ratio	**80/20**	70/30	60/40
Loss	**0.08787**	0.09111	0.09371

**Table 5:** Results of the used optimizers in the classification AlexNet model. Bold indicates the model best result.

Optimizer	Loss
Adam	0.69312
AdamW	0.69310
SGD	**0.08787**

### The proposed hybrid DL-model experiment

The proposed hybrid model’s performance was tested using a testing set. The values and weights of the hyperparameters used in the detection and classification models as determined in the initial experiment were used in this experiment (see Table 6). A loss value of 0.379 loss value was adopted for the DETR model, while the AlexNet model had a loss value of 0.08787. Subsequently, the hybrid model’s performance was tested.

**Table 6:** The best results of the hyperparameter values in detection and classification models.

	DETR detection model	AlexNet classification model
Dataset split	60/40	80/20
Loss	0.379	0.08787
Optimizer	Adam	SGD
Epochs	20	10
Batch size	30	50
Learning rate	0.00002	0.001

**Figure 3:**
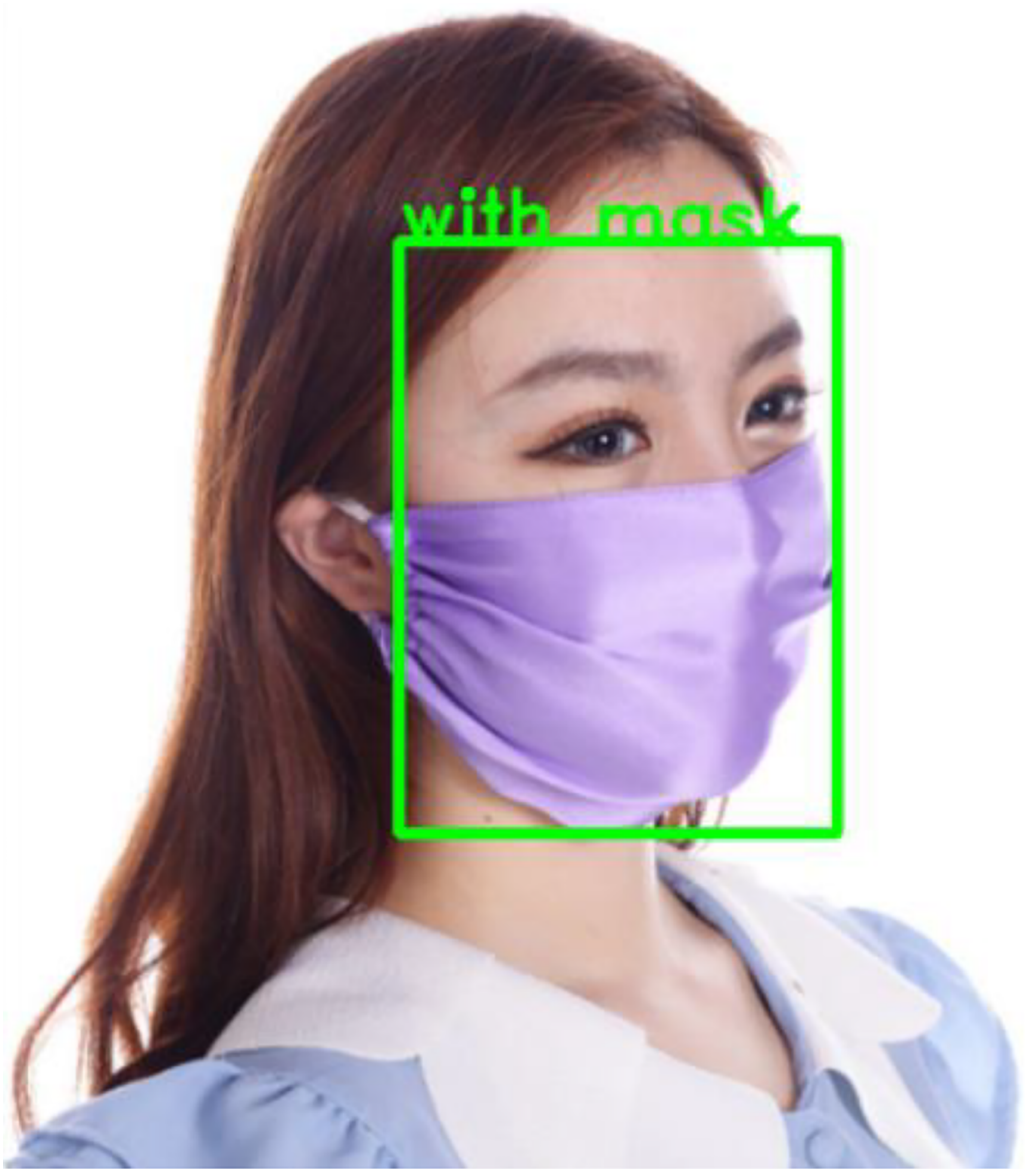
Result of the proposed hybrid model (with mask class). It was resized to 215 * 215 and then to 224 * 224 to be suitable for our models, after that the image was processed inside the models to produce a bounding box around the face objects with a class label (with mask). Image source credits: AIZOO database (https://github.com/AIZOOTech/FaceMaskDetection/blob/master/LICENSE, MIT license) and MAFA database (https://www.kaggle.com/datasets/revanthrex/mafadataset, Apache-2.0).

**Figure 4:**
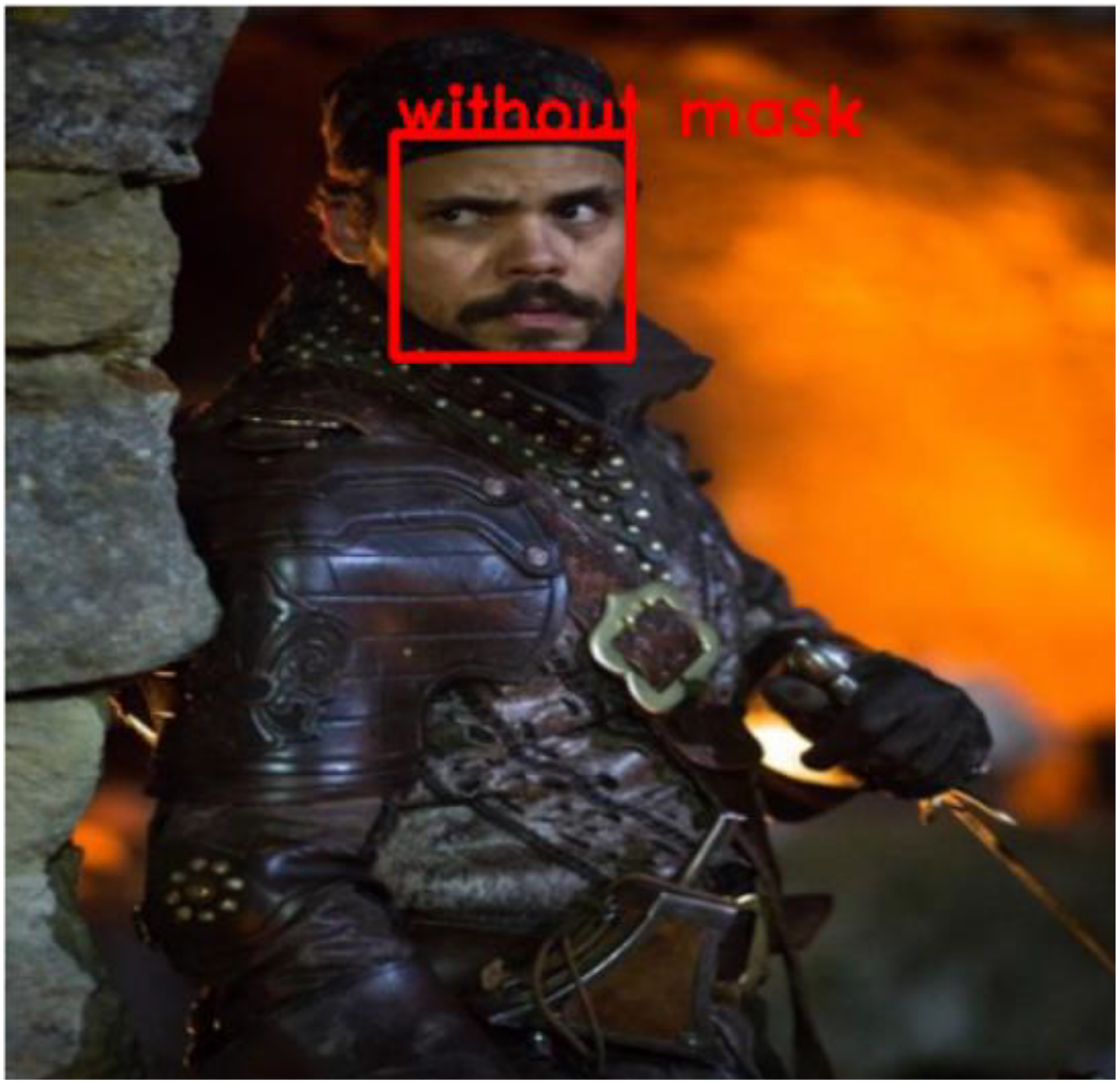
Result of the proposed hybrid model (without mask class). This figure was resized to 215 * 215 and then to 224 * 224 to be suitable for our models, after that the image was processed inside the models to produce a bounding box around the face objects with a class label (without mask). Image source credits: AIZOO database (https://github.com/AIZOOTech/FaceMaskDetection/blob/master/LICENSE, MIT license) and WIDER FACE database (http://shuoyang1213.me/WIDERFACE/index.html, MIT license).

The hybrid model produced bounding box that surrounded the face, and it also provided a class label of either “with mask” or “without mask” for the detected face (see Figs. 3 and 4).

### Discussion of results

The detection model’s training loss decreased significantly for the first 12 epochs, which resulted in a lower level of losses as the model had a loss value of 0.379 in the test. Consequently, the DETR model produced a bounding box around the detected faces, which was fed into the AlexNet model in the final hybrid model in order for it to be classified.

However, the results of the classification model’s test show that the model loss value kept decreasing during both the training and testing processes. As a result, the classification model attained a loss value of 0.08787. Therefore, the training processes for the detection and classification models led to the final hybrid model performing well.

The proposed hybrid model was evaluated using AP, which is a common metric for evaluating DL detection models as it provides the AP of all possible thresholds, thereby allowing for a comparison of the models’ prediction ordering without considering a specific threshold. The testing set’s AP was calculated at 89.4%, thereby indicating the proposed hybrid model’s efficacy.

In addition to the AP metric, another important evaluation criterion for face mask detection tasks is the model execution time, which plays a vital role in real-time tasks. The proposed hybrid model took approximately 2.8 s to classify people into the “with mask” or “without mask” categories. This means that the hybrid model achieved promising results in terms of real-time prediction compared to previous studies.

### Comparison to related studies

The proposed hybrid model was compared to the face mask detection models developed by Loey et al. (2021b) and Ge et al. (2017). All of these models are CNN-based models, with YOLO-v2 and ResNet-50 being used by Loey et al. (2021b), and LLE-CNNs being used in Ge et al.’s (2017) study (Table 7). YOLO-v2 and ResNet-50 were trained using the MMD and FMD datasets, while the LLE-CNN was trained using the MAFA dataset, which is one of the data sources for the AIZOO dataset adopted in this study. All of the previous CNN-based models achieved AP value below 82%. Therefore, this study’s proposed hybrid DL model’s AP value of 89.4% was better than those of Loey et al.’s (2021b) and Ge et al.’s (2017) pure CNN DL models. Moreover, this is the first study to calculate the model’s execution time, which was computed in our study for the purpose of allowing a comparative analysis in future research.

**Table 7:** Comparison with previous work. Bold indicates the model best result.

Models	DL method	AP	Running time
Yolo-v2 and ResNet-50 (Loey et al., 2021b)	CNN	81%	–
LLE-CNNs (Ge et al., 2017)	CNN	76.4%	–
Our proposed hybrid DL model	**Hybrid**	**89.4%**	**2.83 s**

In conclusion, our proposed hybrid model comprising transformer and CNN models provides real-time prediction and high AP rates of 89.4% compared to previous CNN-based face mask detection models.

## Conclusion and future work

Much attention has been paid to face mask detection using DL methods, especially after the COVID-19 pandemic, and some CNN-based models have been proposed to solve this problem. However, there is still a room for improvements in terms of increasing the speed and accuracy of face mask detection. Therefore, this research aims to avoid the tedious and difficult task of manually monitoring people’s behavior in public places and is geared towards enhancing the speed and accuracy of face mask classification and detection. Three DL models were developed herein: the first was used for face detection; the second was used to classify masked and unmasked faces; and the third was a hybrid model comprising the two previous models that was developed to determine whether people were wearing masks.

The proposed hybrid model was developed using DETR and AlexNet algorithms that were used to detect faces in digital images and classify whether people were wearing the face masks, respectively. The models were extensively tested using different hyperparameters and optimization methods. The detection model’s performance was assessed in terms of AP and execution time using the testing set. After evaluating the model, it compared to other face mask detection models that used the AIZOO and MMD datasets. The results for the proposed hybrid model showed an AP of 89.4%, thereby outperforming the other state-of-the-art models. Moreover, the execution time of the proposed hybrid model was approximately 2.8 s, indicating that the proposed model has the potential for real-time detection and can be easily deployed in real applications.

Future research can test the performance of the proposed hybrid model using larger face mask datasets that include rotated faces. Moreover, the proposed hybrid model can be enhanced by ensuring that people’s IDs can also be recognized.

## Supplemental Information

10.7717/peerj-cs.1265/supp-1 Supplemental Information 1 DETR detection model code.

10.7717/peerj-cs.1265/supp-2 Supplemental Information 2 AlexNet classification model code.

10.7717/peerj-cs.1265/supp-3 Supplemental Information 3 The proposed final hybrid model code.

## Additional Information and Declarations

### Data Availability

The following information was supplied regarding data availability: The code is available in the Supplemental Files. The data is available at Figshare: Al-Sarrar, Haifa; Al-Baity, Heyam (2022): AIZOO and MMD datasets. figshare. Dataset. https://doi.org/10.6084/m9.figshare.21554835.v1.
